# Immunosuppressive MDSCs induced by TLR signaling during infection and role in resolution of inflammation

**DOI:** 10.3389/fcimb.2013.00052

**Published:** 2013-09-18

**Authors:** Anuradha Ray, Krishnendu Chakraborty, Prabir Ray

**Affiliations:** ^1^Division of Pulmonary, Allergy and Critical Care Medicine, Department of Medicine, University of Pittsburgh School of MedicinePittsburgh, PA, USA; ^2^Department of Immunology, University of Pittsburgh School of MedicinePittsburgh, PA, USA

**Keywords:** TLR, LPS, bacteria, MDSC, pneumonia, lung, resolution, inflammation

## Abstract

Ligand-mediated activation of toll-like receptors (TLRs) not only induces inflammation but also immune suppression, which is an emerging area of investigation. Multiple negative feedback intracellular mechanisms have been described that are brought into play to prevent uncontrolled TLR activation. However, the identification of TLR-induced regulatory myeloid cells is a relatively recent development that has ramifications in pathogen-induced disease state as well as in cancer. Our efforts to understand how a high dose of lipopolysaccharide (LPS), a ligand of TLR4, suppresses allergic airway inflammation led to the identification of myeloid cells that are CD11b^+^Gri^int^(Ly6G^int^)F4/80^+^ and are phenotypically and morphologically similar to myeloid-derived suppressor cells (MDSCs) which are best studied in the context of cancer. MDSCs have been also detected during infection by various bacteria, parasites and viruses, which can engage different TLRs. These TLR-induced myeloid cells produce different types of mediators to influence immune response and inflammation that can be either beneficial or detrimental to the host. One beneficial function of TLR4/MyD88-triggered MDSCs in the lung is to efferocytose apoptotic neutrophils to help resolve inflammation elicited during bacterial pneumonia. A better understanding of the generation and function of these regulatory cells would be helpful to harness their potential or suppress their function for disease-specific immune regulation.

Microorganism-associated molecular patterns are recognized by pattern-recognition receptors such as Toll-like receptors (TLRs), NOD-like receptors and RIG-I-like receptors in different cells of infected hosts to initiate a rapid innate immune response as the first step in defense against the invading pathogen. The innate immune response, in turn, induces an appropriate adaptive immune response to effectively protect the host with the establishment of a memory response for a rapid recall response during subsequent exposure to the pathogen. After the identification of the toll protein in Drosophila as an essential host-defense mechanism in an organism that lacks the adaptive immune system (Lemaitre et al., 1996), TLR4 was the first TLR to be identified in mammals (Medzhitov et al., 1997; Poltorak et al., 1998; Qureshi et al., 1999). The desire to identify a counterpart to the Drosophila Toll in the mammalian system was largely driven by the need to understand the pathogenesis of bacterial infection-induced sepsis syndrome (Rittirsch et al., 2008). Thus, lipopolysaccharide (LPS), a component of the outer membrane of Gram-negative bacteria, was identified as a ligand of TLR4. Since then, 10 and 12 functional TLRs have been characterized in humans and mice, respectively (Kawai and Akira, 2010). In addition to bacterial LPS, the TLR4 complex is triggered by multiple additional ligands. This process ultimately results in downstream activation of NF-κB or IRF-1 via the adaptors MyD88 and TRIF to induce transcription of cytokine and chemokine genes and a host of other genes that collectively ramp up the host's immune defense mechanisms.

## Regulation of TLR-induced intracellular responses

Although TLR induced pro-inflammatory response is crucial for the elimination of the invading pathogen, uncontrolled immune activation leads to collateral tissue damage. Therefore, stringent negative feedback is necessary to temper TLR-induced signaling in target cells. These measures include a range of extracellular and intracellular decoy receptors, membrane-bound suppressors, intracellular negative regulators, degradation of TLRs, and TLR-induced apoptosis (Liew et al., 2005; Kondo et al., 2012). A distinct TLR-induced immunosuppressive mechanism in different organs, as discovered in some laboratories including our own, is the development of myeloid-derived suppressor cells (MDSCs). In this review, we will discuss the characteristics of MDSCs induced by TLR agonists such as LPS and by pathogens and the emerging field of MDSC-mediated suppression of immune responses.

## Development of a regulatory myeloid cell, MDSC, by LPS-TLR4 signaling to control immune responses

**Figure 1:**
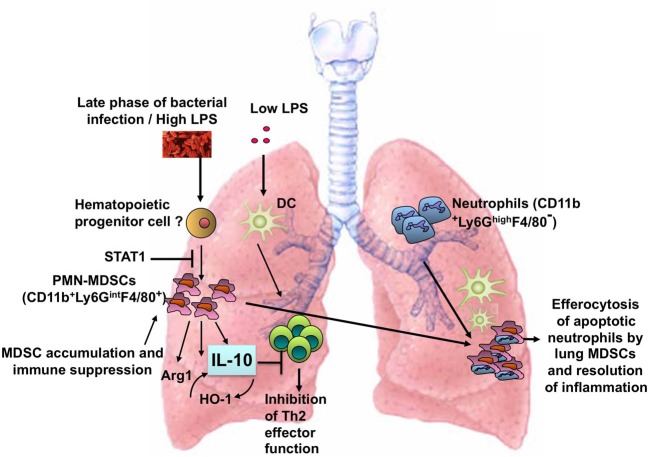
**Induction of MDSCs in the lung in response to a high dose of LPS or bacterial infection**. The lung MDSCs express Arg1 and produce IL-10 and suppress Th2 effector function in the context of allergic airway inflammation and phagocytose apoptotic neutrophils during bacterial pneumonia.

LPS exerts differential effects on immune responses in the lung depending on the dose. In the context of exposure to the model allergen ovalbumin (OVA), a very low dose (< 1 ng) of LPS does not induce adaptive immunity and instead tolerance to OVA develops (Oriss et al., 2005). An intermediate dose (100 ng) of LPS induces a Th2-biased response to OVA and promotes allergic airway inflammation (Eisenbarth et al., 2002). Higher doses of LPS (1–100 μg), however, inhibit allergic inflammation (Gerhold et al., 2002, 2008; Rodriguez et al., 2003; Delayre-Orthez et al., 2005). Our efforts to understand the mechanism(s) by which a high dose of LPS inhibits allergic airway inflammation led to the discovery of a new role for the MDSC as a TLR4/MyD88- but not TRIF-induced cell type that can influence T cell responses in the lung (Arora et al., 2010). Figure 1 illustrates the induction of lung MDSCs in response to LPS or bacterial infection and the function of these cells in the lung tissue.

In mice, MDSCs are uniformly characterized as CD11b^+^Gr1^+^ cells. Since anti-Gr1 recognizes both the Ly6G and Ly6C epitopes, use of antibodies with monospecificity toward one or the other has shown differential expression of these molecules on different types of MDSCs. Typically, CD11b^+^Ly6C^high^ MDSCs are mononuclear and considered to be monocytic (Mo-MDSC) while CD11b^+^Ly6G^+^Ly6C^low/−^ MDSCs consist of multilobed nuclei and represent granulocytic or neutrophilic MDSCs (PMN-MDSC). Additional cell surface molecules including the α-chain of the receptor for IL-4 and IL-13 (CD124), the mouse macrophage-expressed molecule, F4/80, M-CSF-1R or c-*fms* (CD115) and the costimulatory molecule CD80 have also been identified in different combinations on some subsets of MDSCs (Gabrilovich and Nagaraj, 2009; Ostrand-Rosenberg and Sinha, 2009; Peranzoni et al., 2010). LPS-induced MDSCs in the lung have the phenotype CD11b^+^Ly6G^int^Ly6C^low/−^F4/80^+^CD80^+^.

Much before LPS was shown to be a stimulus for MDSC accumulation, multiple factors, associated with chronic inflammation and cancer such as VEGF, GM-CSF, G-CSF, IL-1β, IL6, and PGE2 were described as inducers of MDSCs (Gabrilovich and Nagaraj, 2009). However, the cell surface phenotype and mediator employed by the MDSCs induced by the various types of agents to suppress immune responses are not the same as discussed below.

## LPS-induced lung MDSCs are tissue resident and can suppress effector T cell function

A previous study described proliferation of migratory hematopoietic stem and progenitor cells (HSPCs) within extramedullary tissues in response to LPS (Massberg et al., 2007). Although ~70% of the myeloid cells generated in the presence of LPS expressed the dendritic cell (DC) marker CD11c, a subset of lower frequency (10%) expressed Gr1 at intermediate to high levels and may have been related to MDSCs. In our study, after infusion of GFP^+^ lineage^neg^ (lin^−^) bone marrow progenitor cells into naïve mice, LPS instillation into the lungs promoted the accumulation of GFP^+^CD11b^+^Gr1^int^(Ly6G^int^) cells in the lung tissue (Arora et al., 2010).

LPS-induced Gr1^int^ cells lack CCR7, which is essential for the migration to lymph nodes (Debes et al., 2005). LPS-induced Gr1^int^ cells were not detectable in the lung-draining lymph nodes (LNs) and have been largely identified as tissue-dwelling cells (Arora et al., 2010; Poe et al., 2013). The earlier study also showed that LPS stimulation not only enhances the local proliferation and differentiation of HSPCs but also reduces the migratory capacity of HSPCs within extramedullary tissues by interfering with S1P-S1P1-dependent signaling (Massberg et al., 2007). It was similarly shown *in vitro* that incubation of HSPCs with TLR ligands triggers HSPC proliferation and rapid myeloid differentiation (Nagai et al., 2006). Intradermal injection of *Salmonella typhimurium* or LPS was shown to induce a potent local innate inflammatory response that blocked DC differentiation and migration to the draining LNs (Rotta et al., 2003).

MDSCs are well-known for their ability to inhibit T-cell proliferation and immune responses (Nagaraj et al., 2013). MDSCs utilize multiple mechanisms to exert their suppressive functions and recent studies suggest communication between MDSCs and T cells which is not just limited to cancer but to different types of inflammation-associated conditions which has been comprehensively reviewed recently (Nagaraj et al., 2013). The best-described mechanisms/mediators related to immunosuppression by MDSCs include expression of Arginase 1, nitric oxide, peroxynitrite and reactive oxygen species. MDSCs can also block T-cell activation by deprivation of L-cysteine, an essential amino acid required for T-cell activation and function (Srivastava et al., 2010). Various other mechanisms used by MDSCs to suppress immune responses have also been suggested which include upregulation of cyclooxygenase 2 and prostaglandin E2 (Rodriguez et al., 2005), secretion of TGF-β (Yang et al., 2008; Filipazzi et al., 2012) and induction of Tregs (Huang et al., 2006; Serafini et al., 2008; Nagaraj et al., 2013). Our study showed that the IL-10/Arg1 axis is involved in the suppressive activity of lung MDSCs on Th2 cells (Arora et al., 2010). In another study, HO-1 was implicated in the suppression of alloreactive responses by LPS-induced MDSCs (De Wilde et al., 2009). HO-1 was previously shown to promote IL-10 expression and conversely IL-10 can induce HO-1 expression (Ricchetti et al., 2004; Chauveau et al., 2005). In view of the diversity of immune signals to which MDSCs are exposed in different biological contexts, it is expected that the mechanism of suppression by MDSCs induced by various agents would also vary.

Our study has shown that after repeated exposure of mice to LPS, the MDSCs accumulate with a delayed kinetics in the lung tissue and their numbers increase significantly over baseline only 3 days after LPS instillation once every day (Poe et al., 2013). Since the MDSCs in the lung remain in the tissue after each LPS instillation unlike dendritic cells (DCs) which traffic readily to the LNs (Arora et al., 2010), this differential response causes selective enrichment of the MDSCs over DCs in the tissue (Arora et al., 2010). The LPS/TLR4-induced lung MDSCs suppress the ability of lung DCs to promote Th2 cytokine production, upregulate GATA-3 or induce STAT5 activation in primed Th2 cells, both transcription factors being critical in Th2 effector function (Zhang et al., 1997; Zheng and Flavell, 1997; Zhu et al., 2003). Since STAT5 activation promotes T cell viability (Wofford et al., 2008; Hand et al., 2010), it is possible that lung MDSCs compromise Th2 cell survival thereby reducing the size of the memory T cell pool (Hu et al., 2001; De Wilde et al., 2009). Thus, collectively, it appears that an important effector function of TLR-induced MDSCs is not directed to the LN to influence the development of adaptive immune functions but to control immune responses in the tissue. Of note, flagellin (TLR5)-induced MDSCs have been identified in the lung in the context of cystic fibrosis and associated infection by *Pseudomonas aeruginosa* (Rieber et al., 2012). Although it is proposed that these MDSCs may inhibit T cell responses against the bacteria based on the ability of the cells to inhibit T cell proliferation *ex vivo*, it is unknown whether these TLR5-induced MDSCs indeed traffic to the lung-draining LNs where effects on T cell proliferation would be most relevant. Alternatively, these MDSCs may also function to dampen effector T cell function in the tissue.

## Bacterial pneumonia and resolution of inflammation: a novel role for TLR-induced MDSCs

Given that a high dose of a bacterial component, LPS, was found to be a potent stimulus for MDSC development, it seemed logical to us that bacterial infection of the lung would also trigger MDSC expansion. Toward this end, the response to the Gram-negative bacterium, *Klebsiella pneumoniae*, was studied. *K. pneumoniae* is a common pathogen in hospital-acquired pneumonia, particularly in chronically ill subjects (Neuhauser et al., 2003). Bacterial pneumonia remains a leading cause of morbidity and mortality (Ware and Matthay, 2000; Lee and Downey, 2001; Abraham, 2003; Mizgerd, 2006, 2008; Tsai and Grayson, 2008; Craig et al., 2009; Balamayooran et al., 2010). Three million cases of pneumonia are reported annually in the United States alone, which causes 40,000–70,000 deaths each year (Mizgerd, 2008). *K. pneumoniae* induces massive neutrophil influx followed by extensive lung destruction and TLR4/MyD88 signaling has an important role in host defense against *K*. *pneumoniae* (Chan et al., 2009; Wieland et al., 2011). A number of studies have documented the role of both alveolar macrophages and neutrophils in innate host defense against bacterial pathogens including *K*. *pneumoniae* (Broug-Holub et al., 1997; Craig et al., 2009). However, much less is known about the relationship between these two cell types with regard to bacterial clearance and resolution of lung inflammation. It is critical that neutrophil-associated (immune-mediated) pathology be controlled to prevent collateral damage during defense against the invading pathogen (Lee and Downey, 2001; Abraham, 2003). The problem is, therefore, 2-fold. Bacteria must be cleared and the ensuing host inflammatory mechanisms used for bacterial clearance must also be attenuated to prevent lung tissue damage and high mortality. The significance of this question can be best appreciated in the context of pneumonia precipitating ALI/ARDS (acute lung injury/acute respiratory distress syndrome) in which excessive lung inflammation is an overriding factor of injury suggesting inadequate control by anti-inflammatory mechanisms.

Defense against pathogenic bacteria involves a set of well-orchestrated events in which the first step is recognition of the invading pathogen by the host in which TLRs play an important role. A key purpose of sensing pathogens via TLRs is to rapidly engage an innate immune response to clear the pathogen (Barton, 2008; Soehnlein and Lindbom, 2010). The cells of the innate immune system that are well-characterized with respect to phagocytosis and killing of internalized bacteria are alveolar macrophages and neutrophils (Barton, 2008; Soehnlein and Lindbom, 2010). Typically, alveolar macrophages participate initially but are taken over by neutrophils that are rapidly recruited to the site of infection aided by chemokines, whose major sources are lung epithelial cells and macrophages (Bergeron et al., 1999). Neutrophils generate various noxious products including reactive oxygen species and proteases that are not only harmful for the pathogen but also for the host's own cells (Lee and Downey, 2001; Abraham, 2003; Mizgerd, 2006, 2008; Balamayooran et al., 2010). Therefore, once the pathogen is cleared, the immediate next goal of the host is to mount an appropriate anti-inflammatory response to limit further neutrophil recruitment. Neutrophils also have a relatively short life span and rapidly begin to undergo apoptosis at the site of inflammation. To prevent lung injury, these apoptotic cells need to be rapidly cleared by phagocytes, a process termed efferocytosis. It is at this last phase of the host's response to infection that the MDSCs step in. As observed with LPS exposure, after infection, MDSCs do not accumulate rapidly in the lung but rather develop late after infection (Poe et al., 2013). This makes perfect sense since the lung MDSCs produce IL-10, which if produced too early is detrimental to bacterial clearance since it impedes neutrophil recruitment (Poe et al., 2013). We showed that the lung MDSCs efficiently efferocytose apoptotic neutrophils, which is aided by IL-10 produced by the MDSCs. The successful clearance of pathogens, dampening of neutrophil infiltration and clearance of dead neutrophils ultimately restores tissue homeostasis.

In the context of cancer, MDSCs have been shown to promote the development of regulatory T cells (Tregs) (Nagaraj et al., 2013). Whether this is also true for lung MDSCs during infection remains to be determined. However, Tregs were implicated in suppression of lung inflammation in a model of LPS-induced lung injury (D'Alessio et al., 2009).

## TLR-induced lung MDSC development and the JAK/STAT pathway

GM-CSF has been implicated in innate immune responses in the lung in response to LPS (Bozinovski et al., 2002). *In vitro*, the combination of LPS and GM-CSF induces differentiation of lin-progenitor cells into MDSCs, which suggests co-operation between TLR/MyD88 and Jak2/STAT5 pathways in MDSC development (Arora et al., 2010). LPS-induced MDSCs in the lung produce GM-CSF and IL-6, which have the ability to activate STAT5 and STAT3, respectively, in cells in an autocrine or paracrine fashion. STAT5 activation promotes MDSC survival (Condamine and Gabrilovich, 2011). Jak2/STAT3 signaling has been also shown to play a crucial role in tumor-associated MDSC generation (Nefedova et al., 2004; Condamine and Gabrilovich, 2011). Autocrine IL-6-induced STAT3 signaling downstream of Hsp72/TLR2 was implicated in MDSC-suppressive function (Chalmin et al., 2010). In our studies, neutralization of IL-6 blunted MDSC numbers in *ex vivo* cultures of the cells (Poe et al., 2013). Collectively, these studies suggest that TLR4 activation by a high dose of LPS in precursor cells induces GM-CSF and IL-6 production causing STAT5 and STAT3 activation respectively, which can support the development and function of MDSCs. In keeping with the noted antagonism between STAT1 and STAT3, STAT1-deficient mice were examined in our study for lung MDSC numbers as compared to wild-type mice after bacterial infection. Indeed, lung MDSC numbers almost doubled in STAT1^−/−^ mice in response to bacterial infection with fewer neutrophils as compared to that in wild-type mice (Poe et al., 2013). These observations suggest that increasing MDSC numbers via STAT1 inhibition in combination with antibiotics may be beneficial in the context of non-resolving pneumonia.

## MDSC induction by pathogens VIS-À-VIS use of TLR agonists in cancer

Not only in the lung, in other tissues as well, infection by bacteria, viruses and parasites can promote MDSC accumulation. Table 1 outlines a comparison of MDSCs that have been detected during infection by various pathogens and those that are most commonly associated with cancer, albeit much less information is available about the former. The common inducer in both contexts is inflammation. Upon reviewing currently available information it is evident that an important role of MDSCs is to exercise balance between host-defense-associated inflammation mounted during an immune response and inflammation-mediated tissue pathology (Ortega-Gomez et al., 2013). In the case of sepsis, MDSC development induced by acute phase proteins was shown to be crucial for control of systemic inflammation and sepsis-induced mortality (Sander et al., 2010). While our study shows a beneficial effect of MDSCs in mediating resolution of inflammation during bacterial pneumonia, in the case of infection by influenza virus, MDSC effector function needs to be regulated by iNKT cells in the absence of which immune suppression exercised by MDSCs causes increased viral titer and mortality (De Santo et al., 2008). A recent study has proposed that host susceptibility to co-infection by viruses and bacteria results not due to exaggerated activation of some well-described immune pathways but due to failure to tolerate excessive tissue damage after infection (Jamieson et al., 2013). However, since lung MDSCs have been shown to regulate susceptibility to single infection by either viruses (De Santo et al., 2008) or bacteria (Poe et al., 2013), it is possible that MDSCs regulate both inflammation and tissue damage during co-infection via one or more of its mediators. In this regard, the kinetics of MDSC development post infection (De Santo et al., 2008; Poe et al., 2013) may dictate the ultimate outcome.

**Table 1:** **MDSCs in cancer and infection**.

**MDSC context**	**Species**	**Infectious agent**	**Phenotype**	**Function and mediator(s)**	**References**
Cancer	Mouse		CD11b^+^Ly6C^low^Ly6G^+^CD115^+/−^F4/80^low^CD124^+/−^ (PMN-MDSC)	Reactive Oxygen Species (ROS)	Reviewed in Gabrilovich et al., 2012; Nagaraj et al., 2013
			CD11b^+^Ly6C^+^Ly6G^−^CD115^+^F4/80^+^CD124^+^ (Mo-MDSC)	Arg1, ROS, NO, and Reactive Nitrogen Species	
	Human		CD11b^+^CD33^+^CD14^−^HLA-DR^low/−^CD15^+^ (PMN-MDSC)	Arg1, ROS	Reviewed in Filipazzi et al., 2012; Gabrilovich et al., 2012
			CD11b^+^CD33^+^CD14^+^HLA-DR^low/−^CD15^low/−^ (Mo-MDSC)	TGF-b	
Infection	Mouse	Bacterial product/Bacteria	1. CD11b^+^Ly6G^int^Ly6C^low/−^F4/80^+^CD115^−^CD124^−^ (LPS and *Klebsiella pneumoniae*)	1. (a) Inhibits Th2 function and allergic inflammation and (b) Promotes resolution of inflammation after bacterial pneumonia Mediator: IL-10	1. Arora et al., 2010; Poe et al., 2013
			2. In CF patients and in response to *Pseudomonas aeruginosa* (PMN-MDSCs)	2. Not described	2. Rieber et al., 2012
			CD11b^+^Ly6C^int^Ly6G^−^ (*Mycobacterium bovis*)	Traffics to draining lymph node and inhibits T cell priming but does not kill bacteria Mediator: NO	Martino et al., 2010
			CD11b^+^Gr1^+^ (sepsis)	Inhibits Th1 but promotes Th2 responses in the spleen, controls systemic inflammation and sepsis-induced mortality Mediator: IL-10	Delano et al., 2007; Sander et al., 2010
		Virus	CD11b^+^Gr1^+^F4/80^+^ (Influenza A)	Inhibition of anti-viral immune responses Mediators: Arg1, NO and IL-10	De Santo et al., 2008; Jeisy-Scott et al., 2011
		Parasite	CD11b^+^Gr1^+^	Mediator: NO	Reviewed in Van Ginderachter et al., 2010
			CD11b^hi^Ly6C^hi^F4/80^int^ (*Leishmania major*)	Kills parasites and inhibits T cell proliferation Mediator: NO	Pereira et al., 2011

Similar to our observation of the ability of a combination of GM-CSF and LPS to induce MDSCs *in vitro* (Arora et al., 2010), a combination of IFN-γ and LPS was found to enhance the development and activation of bone marrow-derived MDSCs while simultaneously inhibiting the differentiation of bone marrow cells to DCs (Greifenberg et al., 2009). Not only LPS, but various other danger-associated molecular patterns (DAMPs) such as S100A, and heat shock proteins released from dying cells that also engage TLR4 or other TLRs have the potential to promote accumulation of MDSCs or enhance their function (Cheng et al., 2008; Bunt et al., 2009). Hsp72 from tumor-derived exosomes was shown to promote STAT3-dependent immunosuppressive function of MDSCs in a TLR2/MyD88 dependent manner (Chalmin et al., 2010). Also, the fact that various pathogens that can activate diverse TLRs can also promote MDSCs suggests that the ability to cause MDSC-mediated immunosuppression is not just restricted to TLR4.

Immunosuppression resulting from TLR engagement is of particular importance in cancer therapy. In fact, most anti-cancer strategies involving TLR agonist monotherapy have proven unsuccessful in clinical trials (Guha, 2012). For example, in a pre-clinical study, failure of imiquimod, a TLR7 agonist, in antitumor therapy was associated with IL-10-mediated immunosuppression. IL-10 neutralization significantly enhanced the efficacy of imiquimod (Lu et al., 2010). In contrast, use of TLR agonists in combination with a cancer vaccination approach or chemotherapy has yielded more promising results toward the induction of anti-tumor CTL responses (Ridnour et al., 2013). Most importantly, while TLR signaling and MDSCs portend poor prognosis for cancers (Ridnour et al., 2013), they appear to be beneficial in regulating tissue inflammation in the lung (Arora et al., 2010; Poe et al., 2013) or systemic inflammation during polymicrobial sepsis (Sander et al., 2010).

## Concluding remarks

TLR signaling elicited by pathogens or by components of pathogens in concert with signals imparted by growth factors such as GM-CSF and IFN-γ has the potential to induce MDSCs in different organs including the lung. MDSCs can be either beneficial or detrimental to the host depending on the infectious agent, involved organ and mediator(s) released. High LPS dose or bacterial infection elicits MDSCs in the lung that play an important role in regulating immune response in the context of diverse pulmonary inflammatory conditions such as allergic airway inflammation and bacterial pneumonia. These TLR4-induced MDSCs accumulate with delayed kinetics after high dose LPS exposure or bacterial infection and have features of PMN-MDSCs. The MDSCs can suppress Th2 effector function and efferocytose apoptotic neutrophils and the common mediator involved is IL-10. The net outcome in each case is resolution of inflammation. Thus, a possible therapeutic approach for non-resolving pneumonia is boosting MDSC numbers via STAT1 inhibition in combination with antibiotics where suppression of unremitting inflammation along with complete elimination of the infectious agent is the desired goal. Unquestionably, a fertile area of future research is to understand context-specific function of MDSCs to suppress or stimulate them for therapeutic benefits.

### Conflict of interest statement

The authors declare that the research was conducted in the absence of any commercial or financial relationships that could be construed as a potential conflict of interest.
